# Beyond the Preventive Measures: A Delphi Method-Based Study of the Impact of the COVID-19 Pandemic on the Mental Health of Different Age Groups

**DOI:** 10.3390/medicina60060978

**Published:** 2024-06-13

**Authors:** Eva Sedlašek, Nina Krohne, Polonca Borko, Ives Zemljarič, Robert Masten, Matej Vinko, Diego De Leo, Vita Poštuvan

**Affiliations:** 1 Slovene Centre for Suicide Research, Andrej Marušič Institute, University of Primorska, 6000 Koper, Slovenia; nina.krohne@iam.upr.si (N.K.); polonca.borko@iam.upr.si (P.B.); diego.deleo@upr.si (D.D.L.); vita.postuvan@upr.si (V.P.); 2 Department of Oncology, University Medical Centre Maribor, 2000 Maribor, Slovenia; ives.zemljaric@ukc-mb.si; 3 Department of Psychology, Faculty of Arts, University of Ljubljana, 1000 Ljubljana, Slovenia; robert.masten@ff.uni-lj.si; 4 Mental Health Centre, National Institute of Public Health of Slovenia, 1000 Ljubljana, Slovenia; matej.vinko@nijz.si; 5 Department of Psychology, Faculty of Mathematics, Natural Sciences and Information Technologies, University of Primorska, 6000 Koper, Slovenia

**Keywords:** pandemic, COVID-19, preventive measures, consequences, mental health, demographic groups, qualitative method, quantitative method

## Abstract

*Background and Objectives:* The coronavirus disease 2019 (COVID-19) preventive measures affected various aspects of people’s lives, while also representing an important risk factor for people’s mental health. In the present study, we examined the negative psychological consequences of the preventive measures on people’s mental health and the protective factors that strengthened their mental health and well-being during the pandemic. *Materials and Methods:* A study, using a combination of qualitative and quantitative methods based on a Delphi protocol, was conducted with a sample of Slovenian professionals who worked with people from different demographic groups (i.e., children and adolescents, emerging adults, the adult working population, the elderly) during the pandemic. We conducted (i) a qualitative study involving semi-structured interviews with 11 professionals and (ii) a quantitative study where 73 professionals completed a structured online questionnaire. *Results:* Experts recognized the disruption of informal face-to-face social contacts as the measure with the greatest impact on people’s lives across all groups studied, the effect being particularly evident in relation to individuals’ development period and socio-demographic characteristics. An individual’s ability to adapt to change and emotional support provided by family or other close persons contributed significantly to maintaining mental health and well-being during the pandemic. *Conclusions:* Considering the interplay of various COVID-19-related risk and protective factors for mental health, enabling and promoting the maintenance and development of social relationships (including through alternative pathways) should be a priority aspect of (mental health) intervention for all demographic groups.

## 1. Introduction

The measures and restrictions implemented during the pandemic to prevent and control the coronavirus disease 2019 (COVID-19) affected various aspects of people’s lives. The measures were aimed at containing the spread of the virus SARS-CoV-2, but at the same time represented an important risk factor for people’s mental health [1].

With the outbreak of the COVID-19 pandemic in March 2020, the Slovenian government declared a state of emergency. It implemented various measures to minimize the spread of the virus (lockdown), including closing educational and working organizations and organizing remote schooling and working where possible. Additional measures included self-isolation, strict instructions not to move outside the household (or later outside the municipal boundaries), and curfew. Wearing masks and regular testing were mandatory. The first lockdown took place from March to May 2020 and the second lockdown from October 2020 to April 2021 (with brief periods of limited activities in schools and working organizations). In October 2021, the third lockdown took place, which allowed the operation of schools and working organizations but required social distancing and prevented social gatherings. The measures were relaxed in April 2022 [2,3].

In the initial phase, about up to 1 month after the outbreak of the virus, people reported mild to moderate effects of the pandemic on their mental health [2]. Initially, adults reported having worries and fears related to the uncertainty and nature of the spread of the virus, as well as fears related to contracting the virus [3,4]. Later (approximately after the first month), while pandemic and preventive measures still persisted, fears about the long-term consequences, changes in living conditions, and employment opportunities were reported [4,5]. An increased prevalence of mental disorders such as anxiety, depression, and post-traumatic stress disorder was reported [6,7,8].

The measures taken to combat the pandemic had a far-reaching impact on people’s lives. These include a reduction in daily physical activity, an increase in sedentary lifestyles, prolonged use of digital screens [9], and disrupted sleep patterns [10]. Maintaining social contacts became more challenging as interpersonal interactions shifted to an online environment, which might not have been as familiar, comfortable, or accessible [11]. Additionally, family relationships were impacted. Many individuals from dysfunctional families were forced to spend more time with family members from the same household, which could have represented a source of stress (e.g., more frequent arguments) or resulted in various forms of violence [12]. Emotional loneliness increased [13,14], indicating dissatisfaction with the closest relationship or a lack of intimate attachment to another person (e.g., partner) [15]. The measures taken during this period had an impact on the lives of all people; however, the changes and the consequences they brought for people depended on the demographic and social characteristics of the individual [16].

Children and adolescents have been identified as a higher-risk group during the pandemic, due to their dependence on adults and their specific understanding of the circumstances based on their developmental stage [17,18,19]. The closing of schools has disrupted the education process, which has affected their ability to learn and retain knowledge [18,20]. Additionally, with schools closing, children and adolescents have been deprived of the opportunity for social interaction with their peers and the support system that schools provide for maintaining healthy habits [20]. Therefore, distance learning has affected children’s and adolescents’ social and emotional development [21].

Young people in transition to adulthood (emerging adults), aged between 19 and 29 years [22], were identified as being at higher risk [23]. A change in the study process, loss of part-time or full-time employment, reduced financial stability, changes, and difficulties in maintaining social contacts during the pandemics represented risk factors that contributed to the difficulties in reaching milestones important for young people’s successful transition to adulthood [17]. An increase in feelings of loneliness, depressive symptoms, and anxiety were recognized in this demographic [6].

During the pandemic, the adult working population reported various changes related to their work and home responsibilities [24] and difficulties in balancing work and family life [24,25,26]. During the pandemic, adults were more likely to report feelings of overload and anxiety related to job insecurity, financial insecurity, and family material insecurity [27]. Compared to men, women were more likely to report the presence of depressive symptoms and psychosomatic problems [28].

Due to the more frequent presence of chronic diseases and a weaker immune system, which could lead to more serious health complications or death if infected with the virus, the elderly represented an extremely vulnerable group [17]. To protect themselves from the virus, isolation of older people was strongly recommended, which resulted in many losing the only social contact they had with others [29]. Trabucchi and De Leo [30] explain that the loss of social contact during the pandemic could lead to feelings of loneliness and feelings of being trapped, which was reflected in an increased risk of developing mental health problems [31].

The purpose of the study was to explore the psychological impact of the pandemic and the measures taken to contain the spread of COVID-19 disease on individuals from different vulnerable demographic groups (i.e., children and adolescents, emerging adults, the adult working population, and the elderly), taking the perspective of mental health professionals. We aimed to identify and explore the specific challenges faced by vulnerable individuals during the COVID-19 pandemic and identify protective factors that significantly contributed to strengthening and maintaining their mental health and psychological well-being. Since the beginning of the pandemic, many studies examined the changes and consequences on people’s lives during the COVID-19 pandemic [32]. The present study aimed to summarize the existing findings and provide a comprehensive overview of to-date results. For this purpose, we used combination of qualitative and quantitative method based on a Delphi study protocol, which enables us to obtain and summarize the opinions and insights of a group of experts from various mental health-related fields.

The following research questions (RQs) were proposed:

RQ1: What measures to prevent and control COVID-19 have affected the lives of specific vulnerable demographic group (e.g., children and adolescents, emerging adults, the adult working population, the elderly), and in what ways have these measures impacted them during the COVID-19 pandemic?
RQ2: What were the specific challenges of each vulnerable demographic group during the COVID-19 pandemic?
RQ3: What protective factors contributed to maintaining the mental health and psychological well-being of each specific vulnerable demographic group during the COVID-19 pandemic?

## 2. Materials and Methods

The Delphi method that represented a protocol base for our study is defined as a communication process between experts from different fields of work [33] aiming to obtain the most reliable opinions of a group of experts and to reach a consensus among a group of experts on a specific topic or issue. The method comprises at least two stages of implementation. The first phase, in which the participants provide written answers to open questions, makes it possible to obtain ideas and opinions from experts on the problem being analyzed. In the second phase, the experts evaluate the ideas or opinions according to their importance to reach a consensus [33]. To address our research objectives and questions, we conducted a study based on a Delphi protocol [33] where the first round was implemented in the form of semi-structured interviews (qualitative study). In the second round, a quantitative approach was adopted by using a structured online questionnaire aimed at gathering the opinions of a larger group of experts to generalize the findings from the first phase.

### 2.1. Qualitative Study

#### 2.1.1. Participants

Professionals working with individuals from specific vulnerable demographic groups were invited to participate in our study via email. Of the 34 professionals contacted, 11 took part in the study (10 female and 1 male). The participants come from diverse fields: psychology (36%), psychotherapy (27%), educational work (18%), psychosocial counseling (9%), sociology (9%), public health (9%), youth work (9%), and work with the elderly (9%). Percentages may exceed 100% as some participants come from more than one field. They have worked with individuals from all four demographic groups during the COVID-19 pandemic. The inclusion criteria for participants were good insights and knowledge about the research topic, experiences in working with a specific demographic group, and the ability to participate in an interview. The original list of potential participants included established Slovenian professionals from various fields of work. The experts, representing a general population of Slovenian professionals, were selected on the basis of their expertise as evidenced by their theoretical and applied knowledge and experience in the studied field (e.g., psychologists working with the elderly in care homes, teachers working with children or adolescents, psychotherapists working with individuals from different demographics), as well as their recognition at national and international levels. We used the contact information that is publicly available on their or their organizations’ websites.

#### 2.1.2. Procedure

Before the interview, all participants were asked to sign their informed consent to participate in the study. The interviews were conducted remotely using the online platform, were audio-recorded, and manually transcribed. The average interview duration was 78 min, with the shortest interview lasting 56 min and the longest 96 min. We used the ATLAS.ti 22 software to process and analyze the interviews conducted. The analysis was carried out according to the principles of thematic analysis [34]. Content saturation was reached, i.e., we collected data up to the point where no new content appeared in the statements. All analyses were based on the authentic data we received within the study.

#### 2.1.3. Instruments

The semi-structured interview consisted of questions formulated based on the literature review and related to all three research questions. Before the survey, we provided participants with the definition of each demographic group. The participants were asked to answer questions related to the demographic group they have worked with during the COVID-19 pandemic. The initial questions were formulated and structured similarly for each demographic group (see the example of questions about the emerging adult demographic group). Additional sub-questions were added during the interview process as specific content emerged.

An example of a provided definition and questions about the emerging adult demographic group:

The term “emerging adults” refers to students and other young adults between the ages of 19 and 29. A similar age range has been defined in some other studies.

The COVID-19 pandemic has brought many changes for students and other young adults. Due to job loss, financial instability, moving to home environments, distance learning, and lack of social contact, emerging adults often fail to reach important milestones that are critical to their successful transition into adulthood. In addition, young adults report many mental health issues such as depression, anxiety, loneliness, and stress to a greater extent compared to before the pandemic.

1. Which preventive measures have had the greatest impact on emerging adults and their lives during the COVID-19 pandemic? And why?
2. What (psychological) needs of emerging adults have become particularly evident during the COVID-19 pandemic? And why?
3. What particular challenges have emerging adults faced during the COVID-19 pandemic?
4. What protective factors have helped to maintain psychological well-being and strengthen the mental health of emerging adults during this time? What are the most common internal and external factors? How can they be recognized?

Based on the participants’ detailed responses and the qualitative analysis of the data, a structured questionnaire was created for the second part of our study.

### 2.2. Quantitative Study

#### 2.2.1. Participants

A total of 73 participants participated in the study and answered a series of questions about the demographic group or groups they worked with. A total of 39 (29%) participants answered the questions for the children and adolescent group, 21 (15%) for emerging adults, 36 (26%) for the adult working population, and 28 (20%) for the elderly. A total of 29% of the participants were psychologists, 19% psychotherapists, 16% social workers, 7% pedagogues or social pedagogues, 4% general practitioners, and 3% psychiatrists. A total of 22% of the participants stated “other” as their profession (e.g., occupational therapist, professor, nurse). Other demographic data were not relevant to the results of the study, so we did not collect them in accordance with ethical principles. The data were statistically analyzed using the IBM SPSS Statistics program.

#### 2.2.2. Procedure

The same 34 professionals invited to participate in the qualitative study were invited by email to participate in the quantitative study. In addition, we expanded the list of potential participants by inviting various institutions and their professionals who have worked closely with specific demographic groups during the COVID-19 pandemic, i.e., who were actively working in the studied field (e.g., care homes for the elderly, primary and secondary schools for children and adolescents, psychologists and psychotherapists working with adults). Sampling was based on a snowball system, i.e., all participants contacted were asked to invite their colleagues to take part in the study in order to obtain as many suitable participants as possible. The participants gave their informed consent before completing the questionnaire in the online program 1KA.

#### 2.2.3. Instruments

The questions in the questionnaire were formulated based on the content and specific topics identified in the first phase of the study. The structured questionnaire addressed the following topics: The impact of COVID-19 changes on the fulfillment of individuals’ basic psychological needs, challenges during the COVID-19 pandemic, risk factors affecting individuals’ mental health at that time, and internal and external protective factors helping to cope with mental distress during the COVID-19 pandemic. The participants had to rank the given content and items according to importance or priority, placing one of the items in the next position. The questions addressed these topics for each demographic group studied (children and adolescents, etc.). The participants answered questions about the demographic groups they were working with during the COVID-19 pandemic.

## 3. Results

The results of the qualitative (Section 3.1) and quantitative part (Section 3.2) of the study address each specific demographic group and their needs.

### 3.1. Qualitative Part

Based on the thematic analysis, we identified key topics for each demographic group analyzed.

#### 3.1.1. Children and Adolescents

The participants disclosed that the disruption of informal face-to-face social contact increased children’s and adolescents’ vulnerability by interfering with and altering the process of social skills development, a key aspect of this developmental stage. Inappropriate and maladaptive behavior (e.g., kicking, biting) and a lack of social skills were reported among children in the first and second triads of primary school. This manifested itself in difficulties in social relationships, such as difficulties in establishing and maintaining social contacts with peers or challenges in teamwork.

*“… all the knowledge; someone can catch up on it later. But the emotional aspect, the social aspect… These are aspects of development stages that kids miss out on, and it’s pretty hard to catch up on that.”*

(P10:9)

For adolescents, the interruption of face-to-face contact affords limited opportunities for exploration, thus affecting the process of identity formation and independence. The participants indicate that the successful accomplishment of developmental tasks associated with adolescence has been significantly impaired during the COVID-19 pandemic due to the deprivation of experiences typical of this developmental phase (e.g., first love, dating, entering high school). The role and influence of peer relationships in adolescence were also emphasized. The participants recognized that the disruption of social contact interferes with the process of establishing and maintaining social relationships with peers, which they consider to be an important aspect of the identity formation process.

“*And also, normally, when young people go out on their own, they start to feel a bit independent, and that gaining of independence is also an important part/…/of this adolescence stage. But now, when they were home all day and we had nowhere to go, it feels to me like we took a step back in that regard. They ended up feeling more and more dependent on their parents…*”

(P4:45)

#### 3.1.2. Emerging Adults

In the group of emerging adults, difficulties in maintaining social contact with friends and peers were recognized as an important consequence of the disruption of daily face-to-face contact, significantly affecting the process of independence and identity formation. The participants indicated that emerging adults’ reduced financial independence, resulting from the loss of part-time or first full-time jobs during that period, contributed to the difficult independence process. The process of identity formation was further affected by the lack of opportunities in different areas of the emerging adults’ lives (e.g., personal, academic, and professional). The disruption of face-to-face contacts was also reflected in the difficulty of maintaining social relationships with parents or younger siblings who did not reside at the same address.

“*But humans are really social beings at our core. We need that genuine connection with others. And somehow this really caused a lot of distress among the young people because, on top of everything, that they somehow, I will say, they felt like they’d regressed five, ten steps, because just as they were getting independent, making progress, really somehow starting their adults’ lives, then they had to just somehow let go of all this and go back…*”

(P9:93)

#### 3.1.3. The Adult Working Population

The participants stated that the disruption of daily face-to-face contact due to social distancing measures during the pandemic made it more difficult for adults to maintain relationships with their older children or parents. Care for parents who did not reside at the same address and needed help with everyday tasks became a challenge. The participants recognized this as an important risk factor contributing to the greater vulnerability of adults

“*… who were taking care of other adults, whether it was a partner, a parent, or some other older relatives. For them, I imagine this was a different dynamic. If you’re working from home and at the same time taking care of them.*”

(P6:62)

Additionally, changed and often more demanding care for younger children and their needs was recognized as an important consequence of social distancing and several other preventive measures (e. g. online school), significantly affecting adults’ work–life balance. During the pandemic, parents had to help their younger children with various school tasks (e.g., homework) and ensure uninterrupted online school processes (e.g., help with logging onto the computer). At the same time, they worked from home. Parents often had to work in the afternoon, in the evening, or outside their working hours. The participants reported that parents of young children were more likely to report strong feelings of overload during that time. They emphasized the increased vulnerability of women, who often performed a so-called “dual role” (i.e., working remotely and supervising the child while attending school online).

“*… There was just no time, because of being so overloaded with work, home-schooling kids. So, all of that actually made the day so saturated, on top of keeping up with all the information, that I actually think that even this protective factor was somehow reduced because parents didn’t… there literally was no time, there was no chance for friends to either call each other or meet up…*”

(P9:9)

#### 3.1.4. The Elderly

The participants reported increased vulnerability of the elderly during the pandemic due to reduced social interactions with children and grandchildren who did not reside at the same address. They recognized fewer social interactions as a major risk factor contributing to increased feelings of loneliness in the elderly, as their already limited social interactions were further reduced or absent. The generally poorer digital literacy of the elderly was recognized as an important factor contributing to the difficulties in maintaining social interactions during that time.

The participants recognized the disruption of daily face-to-face contact, along with some other preventive measures (e.g., mobility restrictions), as important risk factors that contributed to increased vulnerability of the elderly during the pandemic, as they limited the ability to provide instrumental support for the elderly’s daily needs. Individuals who were already dependent on daily assistance to meet their basic needs before the pandemic (e.g., help from family members or community nurses) were considered particularly vulnerable.

“*… And then we have those elderly individuals who are just not good with technology./…/So, again we have this gap here, between the ones who could maintain their social interactions, at least with telephone calls or text messages and so on. And those who, because they are not familiar with technology, they had problems in this aspect or rather it was a case of even greater loneliness because of all this.*”

(P9:57)

#### 3.1.5. Protective Factors

The individual’s ability to adapt effectively to change as an internal protective factor and emotional support from family as an external protective factor were identified as the two protective factors that contributed most to strengthening and maintaining the mental health of individuals from all four demographic groups studied. The participants found that those who were more likely to adapt successfully to change were also more likely to emerge from the situation without major difficulties or distress. Emotional support from close family members contributed greatly to successfully coping with change during that time. This support was reflected in the help individuals received to understand and accept the changes during that time, as well as the support and help they received in coping with the distress and intense emotions that were more common during the pandemic.

“*… A close family as the first, but even if not a closer family, it seems to me that this contact with another human being is what is sincere. That genuine contact is present. It seems to me that this is also something that represents a safe harbor. When you have many problems, but you have the opportunity to talk about them or process these things in another way. This close social network is something that is a good protective factor for a person.*”

(P6:19)

### 3.2. Quantitative Study

Table 1 shows the results of the quantitative part of the study, which includes participants’ opinions on (a) the importance of certain preventive measures in terms of affecting basic human psychological needs, (b) the long-term impact of various challenges on individuals’ mental health, and (c) the importance of various internal and external protective factors for strengthening or maintaining individuals’ mental health and psychological well-being during the pandemic. The results show the percentage of participants who recognized a particular item as the most important and its mean score. An item’s higher percentage or lower mean score was interpreted as greater agreement with the importance of that item. A non-parametric Friedman test was used to account for significant differences between items in specific groups of items for all demographic groups.

**Figure 1:**
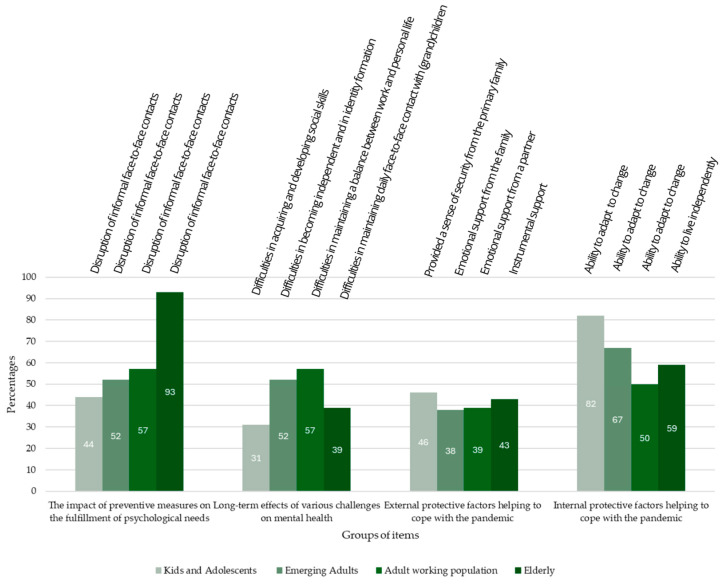
Item from Each Group of Items Recognized as Having the Greatest Impact among Each Different Demographic Group.

Table 1 and Figure 1 show that the highest percentage of participants identified the disruption of informal face-to-face contact as the preventive measure with the greatest impact on the fulfillment of basic psychological needs among emerging adults (52%), the adult working population (57%), and the elderly (93%). Among children and adolescents, the highest percentage of participants (in both cases 44%) identified disruption of face-to-face contact and disruption of the traditional face-to-face school process as the preventive measure with the greatest impact on the fulfillment of basic psychological needs. In addition, significant differences (*p* < 0.001) were found between the items relating to the impact of preventive measures on the fulfillment of psychological needs for all four demographic groups, suggesting the items were recognized as of significantly different importance for each demographic group. A high level of agreement was found among participants regarding internal protective factors to strengthen and maintain an individual’s mental health. In addition, Table 1 and Figure 1 show that the highest percentage of participants identified the individual’s ability to successfully adapt to change as the factor with the greatest impact in children and adolescents (82%), emerging adults (67%), and adults (50%). Significant differences (*p* < 0.001) related to internal protective factors were observed in the demographic group of children and adolescents, emerging adults, and the adult working population.

Several different factors were recognized as important external protective factors. Within each demographic group, there was less agreement among participants regarding the importance of a particular external factor.

Figure 1 shows a visual pattern that confirms the important role of disruption of face-to-face contacts as well as recognizing the ability to adapt to change as one of the key protective factors in coping with challenges and the situation during the COVID-19 pandemic.

### 3.3. Integration of the Results

Social distancing was recognized as the preventative measure with the greatest impact on the lives of people from all four demographic groups (in relation to RQ1), as it manifested and reflected in the disruption of daily face-to-face contact and affected daily life and social interactions across all groups. Each demographic group therefore faced unique challenges and psychological distress, but all were closely related to the individual’s social (and developmental) needs (in relation to RQ2). The importance of social aspects was also evident in the category of protective factors, as emotional support from family or other significant loved ones was recognized as the most important external protective factor in all four demographic groups (in relation to RQ3).

**Figure 2:**
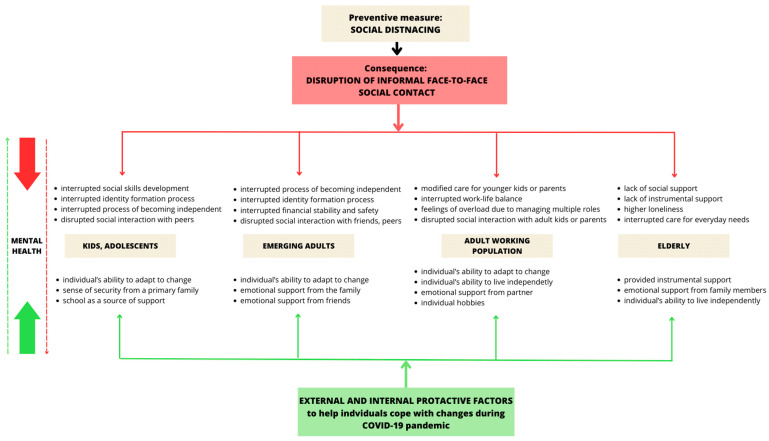
The Mental Health of Specific Demographic Groups during the COVID-19 Pandemic as a Result of the Interplay between the Disruption of Informal Face-to-Face Contact and Various Protective Factors.

The triangulation of both rounds of our study shows the multidimensional impact of the COVID-19 pandemic on different demographic groups, highlighting both common and unique vulnerabilities and protective factors across all four demographic groups (as seen in Figure 2).

The proposed model (Figure 2) summarizes the results of the qualitative and quantitative parts of the study, which suggest that the disruption of informal face-to-face contact had a significant impact on the mental health and psychological well-being of individuals from all four demographic groups, with the nature of (psychological) distress and the consequences differing for each vulnerable group. The differences between the groups were related to the characteristics of the individuals’ age development period and the inability to meet the (developmental) needs typical of a given period.

## 4. Discussion

Based on the results of our study, we recognize pandemic-related psychological vulnerability as an interaction of different risk factors (i.e., factors that threaten our mental health) and protective factors (i.e., factors that preserve and strengthen our mental health). A key risk factor that affected people’s lives during and after the pandemic (including individuals from all demographic groups that we have focused on in the present study) is the so-called disruption of informal face-to-face contacts, a result of social distancing measures and the complete lockdown of the country.

The ban on regular face-to-face social interactions during the COVID-19 pandemic changed the way people spent their leisure time with their friends, peers, relatives, family members, or partners who did not reside in the same place at that time. This had a significant impact on people’s mental health and psychological well-being.

### 4.1. Children and Adolescents

The majority of participants associated the disruption of daily face-to-face interactions among children and adolescents primarily with the interruption of the process of completing the developmental tasks specific to this developmental stage, as the preventive measures reduced the opportunities for the experiences typical and crucial for this developmental stage, including the socialization process. This is consistent with the results of another study from Slovenia [21]. A study from England [35] also found that the lockdown significantly impaired the development of social skills in adolescents.

The participants further emphasized the important role of the difficulties in maintaining social interactions due to the increased influence of peer relationships during this time. The social environment has a significant influence on the social development of adolescents, the development of their self-concept, and their mental health [36]. Adolescence, which is already a sensitive period for an individual’s social development [37], was therefore exposed to additional challenges. Marjanovič Umek and Zupančič [38] recognize identity formation as an important process during adolescence. In addition to the development of other important abilities, skills, or constructs (e.g., cognitive development, emotional development, etc.), identity formation requires the acquisition of a sense of independence. Meanwhile, our findings suggest that the disruption of face-to-face social contact had an impact on adolescents’ attainment of feelings of independence and on the process of identity formation, as there were fewer opportunities to have the experiences typical of adolescence (e.g., falling in love, spending most of their time with friends or peers, graduating from high school). Adolescents had fewer opportunities to explore their environment, take risks, learn and grow, and become independent. According to a study conducted in England [35], the COVID-19 lockdown period has proven to be a significant obstacle in the process of adolescents’ identity formation.

### 4.2. Emerging Adults

Identity formation continues and also takes place during the transition from adolescence to adulthood [39]. During this time, emerging adults develop their identity by exploring different personal life options and gaining psychological and financial independence from their original family [22]. Achieving financial independence is an important criterion for entering adulthood [40]. The results of our study show that maintaining financial stability and security was more difficult during the pandemic, leading to various fears and worries among emerging adults. Several authors have reported similar findings [7,22,39].

A stable social environment, another important aspect of personal development and identity formation during the transition from adolescence to adulthood [41], was changed and challenged by the pandemic and preventive measures. The participants identified difficulties in maintaining daily face-to-face contact with friends and peers as the second most important challenge with long-term effects on the mental health of emerging adults. They emphasized that changes in opportunities to socialize may be reflected in a decreased sense of social support and/or increased feelings of loneliness in this demographic group. Our findings are consistent with the findings of several other studies reporting increased levels of loneliness and an increase in symptoms of depression, anxiety, and post-traumatic stress disorder in young people transitioning into adulthood during the pandemic [6,7,8].

### 4.3. The Adult Working Population

Our findings suggest that the intertwining between the disruption of daily face-to-face contact and the introduction of remote working, which many workers experienced during the pandemic, significantly affected the lives of the adult working population during the pandemic. The difficulty of maintaining social contact among adults was particularly evident in changed and difficult possibilities of maintaining contact with their adult children or their parents who did not live at the same address. A study conducted in Switzerland [8] also found increased stress in adults due to the inability to spend time with family or friends. Our participants emphasize that the disruption of social interactions had a particular impact on the altered and often difficult ability of adults during the pandemic to care for their older parents who required some form of assistance with their daily functioning. The latter contrasts with the findings of a study from Slovenia [42] in which caring for a close older family member during the COVID-19 pandemic is cited as a less important factor in the development of various unpleasant emotions.

Our study identifies the difficulty of maintaining a work–life balance as the main challenge with long-term effects on the mental health of adult workers during the COVID-19 pandemic. Other authors report similar findings [24,25]. The changed and often increased care of children during this time was identified as the most important factor contributing to a more challenging work–life balance for adults. Our results show that the disruption of daily face-to-face social contacts, together with the introduction of remote working, contributed to working adults feeling overwhelmed due to the so-called “dual role” (the role of an employee and the role of a parent). Other authors came to similar conclusions [24]. Griffith [25] points out that many factors typical of the time of the pandemic and faced by adults (e.g., job loss, working from home, caring for children’s schooling, completing work tasks outside working hours…) could lead to burnout and psychological distress during this time. According to our research, women were more often confronted with private (e.g., running a household, helping a child with schooling…) and professional obligations during the pandemic. They were more often forced to reconcile work and private or family life, a challenge that could lead to psychological distress. The participants considered women to be particularly vulnerable during the COVID-19 pandemic. Our findings align closely with a quantitative study from Slovenia [28] that examined the mental health differences in men and women during the COVID-19 pandemic.

### 4.4. The Elderly

The results suggest that the disruption of daily personal social contact had a significant impact on various aspects of the elderly’s lives. Difficulties in maintaining regular contact with children and grandchildren were identified as one of the main consequences of the disruption of face-to-face social interactions in this demographic group. The participants reported that such difficulties may have a longer-term impact on the mental health of older people living alone than those living in care homes. The participants indicated that the ban on face-to-face social contact was reflected in a decrease in social interactions and an increase in loneliness. Similar results have been reported previously, indicating that the strict recommendations to protect the physical health of older people led to an additional reduction in their social contacts and an increase in feelings of loneliness [29,30,43]. Santini et al. [44] reported an increased prevalence of feelings of social disconnection and symptoms of anxiety and depression in older people during the COVID-19 pandemic.

The participants identified less familiarity with technology and its associated applications as an important aspect contributing to older people’s difficulties in maintaining social contacts during the COVID-19 pandemic. Technology helped most other vulnerable groups to maintain social interactions with others during the pandemic. The lower technological literacy of the elderly, which may hinder the maintenance of social contacts via various online tools, is reported by Armitage and Nellums [29] who emphasize the unequal opportunities in accessing technological and online tools. This aspect was not considered in the present study. In the future, it would be worthwhile to investigate the impact of unequal access opportunities and knowledge of technological and online tools on maintaining social contacts during the pandemic in Slovenia. The participants noted that the disruption of face-to-face contact made it difficult to provide instrumental support to older people who needed help with care or daily tasks during the pandemic. Santini et al. [44] reported that older people who were already dependent on family or friends or other forms of support (e.g., home care) before and during the pandemic may develop feelings of worthlessness and of being a burden.

### 4.5. Protective Factors

Our study found that various protective factors (e.g., the school environment as a source of support for children, emotional peer support for emerging adults, and instrumental support for the elderly) were important for maintaining and improving mental health among different demographic groups during the COVID-19 pandemic. Our findings are consistent with Biggs and colleagues’ definition of coping and responding to stress. According to the authors [45], it is important to recognize that the response to a crisis and coping with stress as a subjective experience varies from person to person. The authors define the individual experience of a stressful situation as the result of a complex interaction of various factors (e.g., characteristics of the social environment, functioning of biological processes, personality traits of the person). These represent buffer factors that help the individual to cope with stress more successfully and reduce the risk of negative effects on the individual’s mental or physical health.

Our study shows that emotional support from family and an individual’s ability to adapt to change were the most important factors in maintaining mental health and psychological well-being across all four demographic groups. The role of emotional support from a partner was additionally identified as an important external protective factor in adults, while in children (particularly younger children) it was the sense of security provided by the primary family. The ability to adapt to change, which is known to develop under the influence of individual and environmental factors [46], was identified as an internal protective factor and an important aspect of resilience.

### 4.6. Strengths and Limitations

The use of qualitative and quantitative methods enabled the study to be conducted using a combination of qualitative and quantitative research methods, providing a deep and comprehensive insight into the studied problem. The study contributed to a more detailed understanding of the impact of the pandemic and the preventive measures taken to contain the spread of COVID-19, especially the measure of social distancing of people from different demographic groups. Our study highlights the importance of social interactions as a protective factor that helped to maintain mental health and psychological well-being during the pandemic.

The present study has several limitations posing certain constraints on generalizability. The limitations include the small sample size, especially the small sample of the quantitative part of our study. Potential biases in answering the research questions also require great caution in generalizing the results to a specific demographic group. While the method used allowed for a thorough review of the studied problem, it is also important to be aware of the possible influence of the experts’ personal beliefs when participating in both parts of the study.

The study provides important insights for further work in the field of studying the long-term psychological consequences of the pandemic. Future research directions need to focus on examining the subjective experiences of individuals from the demographic groups studied. This would represent an important extension of the findings of this study.

## 5. Conclusions

By recognizing the different needs, challenges, and consequences that individuals of different demographic groups faced during and after the pandemic, our study highlights the complexity and diversity of the consequences of the pandemic and the preventive measures taken during the pandemic. Furthermore, the study underlines the importance of considering our findings in further studies on the long-term consequences of the COVID-19 pandemic.

Disruption of informal daily face-to-face contact was identified as the most important risk factor for the mental health of individuals across all demographic groups studied in the present study. It was reflected in different consequences and different forms of psychological distress in the different demographic groups studied. Differences in the forms of psychological distress and consequences were found depending on the characteristics of the person’s age development period and the inability to fulfill the (psychological) needs typical for a certain period. The disruption of informal face-to-face contact made it difficult for younger children to acquire social skills, while for adolescents and young people in transition to adulthood, it affected the process of identity formation. Together with other measures, this made it more difficult for adults to maintain a work–life balance. For the elderly, the preventive measures contributed to a reduction in social and instrumental support on the one hand and increased feelings of loneliness on the other. During the pandemic, the individual’s ability to successfully adapt to the changes and emotional support from the close family or other close person contributed most to strengthening mental health and maintaining psychological well-being across all demographic groups.

Considering the interplay of various COVID-19-related risk and protective factors for mental health, with the disruption of daily face-to-face contact emerging as a key challenge for all demographic groups (which also impacts other aspects of people’s lives) and emotional support from family or significant others identified as a crucial external protective factor, promoting and enabling the maintenance and development of social relationships (including through alternative pathways) should be a priority in mental health interventions for all demographic groups.

According to our findings, intervention activities are required at various levels, including promotion, prevention, treatment, and maintenance. In particular, they should focus on promoting and raising awareness of the importance of social relationships and support, especially in times of increased stress, such as the COVID-19 pandemic. In addition, organizing interventions tailored to the specific needs of different population groups can enhance their effectiveness. The study highlights the need for policies that promote social cohesion and provide resources for national or community programs that facilitate social interactions, including outreach programs for different demographic groups, as well as offer emotional support. Further research into the long-term effects of social isolation and the effectiveness of different methods of making connections is needed, using interdisciplinary approaches to develop comprehensive solutions.

## Figures and Tables

**Table 1:** Results of Quantitative Part of Delphi Method-based Study.

Item	Percentage of Professionals ^1^	*M* ^2^ (min–max)	*SD*	*χ* ^2^	*df*
**CHILDREN AND ADOLESCENTS (*N* = 39)**					
**The impact of preventive measures on the fulfillment of psychological needs**				18.67 **	2
Interruption of organized leisure activities	13	2.56 (1–3)	0.72		
Disruption of informal face-to-face contacts	**44**	**1.69** (1–3)	0.70		
Closing of schools (interruption of face-to-face learning)	44	1.74 (1–3)	0.75		
**Long-term effects of various challenges on mental health**				38.77 **	4
Difficulties in maintaining daily face-to-face contact with peers	23	2.67 (1–5)	1.34		
Difficulties in becoming independent and in identity formation	13	3.46 (1–5)	1.25		
Difficulties in acquiring and developing social skills	**31**	**2.31** (1–5)	1.20		
Difficulties in maintaining physical fitness	5	4.15 (1–5)	1.13		
Difficulties in following the school process	28	2.41 (1–5)	1.27		
**External protective factors helping to cope with the pandemic**				68.52 **	5
Emotional support from the family	15	2.46 (1–6)	1.14		
Provided a sense of security from the primary family	**46**	**2.13** (1–6)	1.34		
Accepting attitudes of the primary family (pandemic, measures)	26	3.10 (1–6)	1.64		
Emotional support from friends and peers	3	3.97 (1–6)	1.27		
Emotional support from teachers and other school workers	10	4.46 (1–6)	1.75		
Individual hobbies	0	4.87 (1–6)	1.11		
**Internal protective factors helping to cope with the pandemic**				16.03 **	1
Ability to adapt to change	**82**	**1.18** (1–2)	0.39		
Ability to perform tasks independently (e.g., homework)	18	1.82 (1–2)	0.39		
**EMERGING ADULTS (*N* = 21)**					
**The impact of preventive measures on the fulfillment of psychological needs**				22.32 **	4
Interruption of organized leisure activities	5	3.14 (1–5)	1.15		
Disruption of informal face-to-face contacts	**52**	**1.71** (1–5)	1.01		
Closing of faculties (interruption of face-to-face courses)	29	2.86 (1–5)	1.49		
Introduction of remote working	5	3.90 (1–5)	1.18		
Changed market needs and opportunities	9	3.38 (1–5)	1.32		
**Long-term effects of various challenges on mental health**				21.41 **	5
Difficulties in maintaining daily face-to-face contact with peers	19	2.71 (1–6)	1.49		
Difficulties in maintaining daily face-to-face contact with parents	10	4.57 (1–6)	1.57		
Difficulties in maintaining daily face-to-face contact with a partner	5	5.73 (1–6)	1.29		
Difficulties in following the study process	0	3.52 (1–6)	1.37		
Difficulties in becoming independent and in identity formation	**52**	**2.38** (1–6)	1.77		
Difficulties in maintaining a balance between work and personal life	14	4.24 (1–6)	1.81		
**External protective factors helping to cope with the pandemic**				13.60 *	5
Emotional support from the family	**38**	**2.81** (1–6)	1.75		
Financial support from the family	5	3.86 (1–6)	1.59		
Accepting attitudes of the primary family (pandemic, measures)	10	4.52 (1–6)	1.83		
Emotional support from peers	24	2.90 (1–6)	1.64		
Emotional support from a partner	10	3.10 (1–6)	1.22		
Individual hobbies	14	3.81 (1–6)	1.69		
**Internal protective factors helping to cope with the pandemic**				29.80 **	3
Ability to find meaning in the situation	28	2.00 (1–4)	0.84		
Ability to adapt to change	**67**	**1.52** (1–4)	0.93		
Ability to perform tasks independently (e.g., cooking)	5	3.05 (1–4)	0.87		
Ability to provide financial security	0	3.43 (1–4)	0.68		
**ADULT WORKING POPULATION (*N* = 36)**					
**The impact of preventive measures on the fulfillment of psychological needs**				21.07 **	3
Disruption of informal face-to-face contacts	**57**	**1.73** (1–4)	0.96		
Introduction of remote working	21	2.59 (1–4)	1.12		
Changed market needs and opportunities	19	2.59 (1–4)	1.11		
Mobility restrictions	3	3.08 (1–4)	0.92		
**Long-term effects of various challenges on mental health**				12.34 *	3
Difficulties in maintaining daily face-to-face contact with parents	19	2.51 (1–4)	1.10		
Difficulties in maintaining daily face-to-face contact with adult children	11	2.73 (1–4)	0.93		
Difficulties in maintaining daily face-to-face contact with a partner	13	2.86 (1–4)	1.11		
Difficulties in maintaining a balance between work and personal life	**57**	**1.89** (1–4)	1.15		
**External protective factors helping to cope with the pandemic**				32.24 **	5
Emotional support from a partner	**39**	**2.36** (1–6)	1.55		
Financial support from close family members	3	4.72 (1–6)	1.39		
Accepting attitudes of close family members (pandemic, measures)	11	3.81 (1–6)	1.87		
Emotional support from friends	0	3.72 (1–6)	1.23		
Emotional support from an extended family	22	3.28 (1–6)	1.60		
Individual hobbies	25	3.11 (1–6)	1.70		
**Internal protective factors helping to cope with the pandemic**				20.98 **	4
Ability to adapt to change	**50**	**2.11** (1–5)	1.37		
Ability to live independently	23	3.67 (1–5)	1.37		
Ability to organize effectively	19	2.89 (1–5)	1.39		
Ability to provide financial security	11	3.44 (1–5)	1.25		
Ability to think critically	17	2.89 (1–5)	1.28		
**ELDERLY (*N* = 28)**					
**The impact of preventive measures on the fulfillment of psychological needs**				39.93 **	2
Interruption of organized leisure activities	0	2.11 (1–3)	0.32		
Disruption of informal face-to-face contacts	**93**	**1.11** (1–3)	0.45		
Mobility restrictions	7	2.79 (1–3)	0.57		
**Long-term effects of various challenges on mental health**				1.14	2
Difficulties in maintaining daily face-to-face contact with (grand)children	**39**	**1.86** (1–3)	0.80		
Difficulties in protecting oneself from virus infection	25	2.14 (1–3)	0.80		
Difficulties in accessing medical care	36	2.00 (1–3)	0.86		
**External protective factors helping to cope with the pandemic**				51.54 **	6
Emotional support from a partner	14	4.11 (1–7)	2.10		
Emotional support from family members	**22**	**2.46** (1–7)	1.07		
Emotional support from friends	7	3.82 (1–7)	1.77		
Financial support from family	0	6.04 (1–7)	1.29		
Own financial security	7	4.54 (1–7)	1.90		
Instrumental support	**43**	**2.71** (1–7)	1.80		
Individual hobbies	7	4.32 (1–7)	1.72		
**Internal protective factors helping to cope with the pandemic**				7.63 *	2
Ability to adapt to change	30	2.07 (1–3)	0.83		
Ability to live independently	**59**	**1.59** (1–3)	0.80		
Ability to organize effectively	11	2.33 (1–3)	0.68		

^1^ Percentage of experts = percentage of experts who chose a particular item as the most important or having the greatest impact (using a ranking method), the highest value for each category is in bold; ^2^ *M* = average value of each item (the average rank of importance or impact—the lower the value, the more important the item was or the greater its impact), the lowest value for each category is in bold; * *p* < 0.05, ** *p* < 0.001.

## Data Availability

The data presented in this study are available on request from the corresponding author.
